# DeePred-BBB: A Blood Brain Barrier Permeability Prediction Model With Improved Accuracy

**DOI:** 10.3389/fnins.2022.858126

**Published:** 2022-05-03

**Authors:** Rajnish Kumar, Anju Sharma, Athanasios Alexiou, Anwar L. Bilgrami, Mohammad Amjad Kamal, Ghulam Md Ashraf

**Affiliations:** ^1^Amity Institute of Biotechnology, Amity University Uttar Pradesh, Lucknow, India; ^2^Department of Applied Science, Indian Institute of Information Technology Allahabad, Prayagraj, India; ^3^Department of Science and Engineering, Novel Global Community Educational Foundation, Hebersham, NSW, Australia; ^4^AFNP Med Austria, Vienna, Austria; ^5^Department of Entomology, Rutgers, The State University of New Jersey, New Brunswick, NJ, United States; ^6^Deanship of Scientific Research, King Abdulaziz University, Jeddah, Saudi Arabia; ^7^Institutes for Systems Genetics, Frontiers Science Center for Disease-Related Molecular Network, West China Hospital, Sichuan University, Chengdu, China; ^8^King Fahd Medical Research Center, King Abdulaziz University, Jeddah, Saudi Arabia; ^9^Department of Pharmacy, Faculty of Allied Health Sciences, Daffodil International University, Dhaka, Bangladesh; ^10^Enzymoics, Hebersham, NSW, Australia; ^11^Novel Global Community Educational Foundation, Hebersham, NSW, Australia; ^12^Pre-Clinical Research Unit, King Fahd Medical Research Center, King Abdulaziz University, Jeddah, Saudi Arabia; ^13^Department of Medical Laboratory Sciences, Faculty of Applied Medical Sciences, King Abdulaziz University, Jeddah, Saudi Arabia

**Keywords:** blood-brain barrier, convolutional neural network, deep learning, machine learning, prediction, CNS-permeability

## Abstract

The blood-brain barrier (BBB) is a selective and semipermeable boundary that maintains homeostasis inside the central nervous system (CNS). The BBB permeability of compounds is an important consideration during CNS-acting drug development and is difficult to formulate in a succinct manner. Clinical experiments are the most accurate method of measuring BBB permeability. However, they are time taking and labor-intensive. Therefore, numerous efforts have been made to predict the BBB permeability of compounds using computational methods. However, the accuracy of BBB permeability prediction models has always been an issue. To improve the accuracy of the BBB permeability prediction, we applied deep learning and machine learning algorithms to a dataset of 3,605 diverse compounds. Each compound was encoded with 1,917 features containing 1,444 physicochemical (1D and 2D) properties, 166 molecular access system fingerprints (MACCS), and 307 substructure fingerprints. The prediction performance metrics of the developed models were compared and analyzed. The prediction accuracy of the deep neural network (DNN), one-dimensional convolutional neural network, and convolutional neural network by transfer learning was found to be 98.07, 97.44, and 97.61%, respectively. The best performing DNN-based model was selected for the development of the “DeePred-BBB” model, which can predict the BBB permeability of compounds using their simplified molecular input line entry system (SMILES) notations. It could be useful in the screening of compounds based on their BBB permeability at the preliminary stages of drug development. The DeePred-BBB is made available at https://github.com/12rajnish/DeePred-BBB.

## Introduction

**FIGURE 1:**
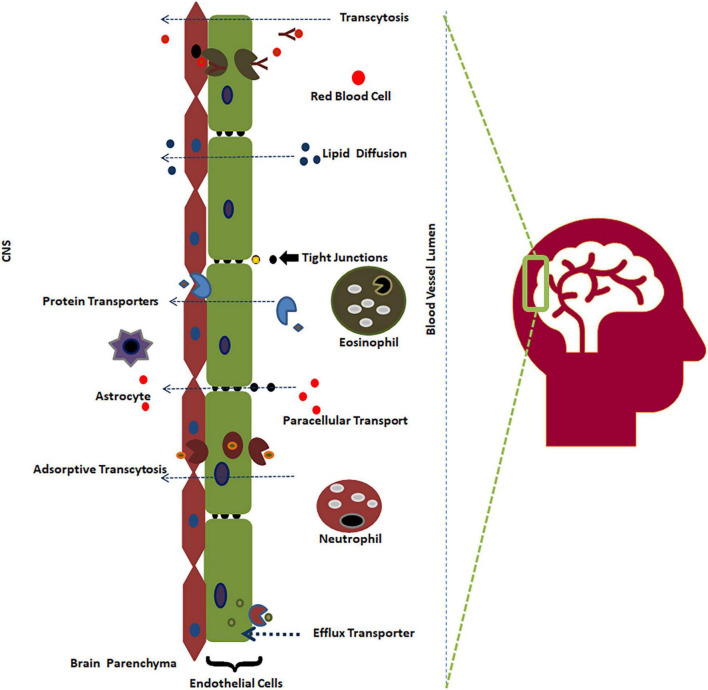
The BBB and its permeability mechanism (Saxena et al., 2021).

Neurological diseases are among the most predominant health issues, with an approximately 28% prevalence in all age groups of patients (Menken et al., 2000). Despite a decrease in communicable neurological diseases, the number of deaths due to neurological diseases has increased to 39% in the last three decades (Feigin et al., 2020). This substantial increase in the absolute number of patients indicates that available therapeutics are scarce to prevent and manage neurological diseases in the current changing global demography. Therefore, it is imperative to find novel and effective therapeutics to target the central nervous system (CNS) to meet the challenges of the ever-increasing absolute number of patients with neurological diseases. An alternative method targeting the molecular and signaling mechanisms at BBB rather than the traditional approaches has become the recent trend in drug target validation (Salman et al., 2022). Drugs must cross the blood-brain barrier (BBB) to act on the CNS. There is a higher attrition rate of drug candidates failing in clinical research due to non-permeability to the BBB compared to potency issues (Dieterich et al., 2003; Pardridge, 2005; Saunders et al., 2014; Hendricks et al., 2015). The BBB is a semipermeable and selective boundary that maintains the steady state of the CNS by protecting it from external compounds (98%) (Figure 1; Pardridge, 2002). As drugs need to enter the CNS to impart therapeutic activity, it becomes crucial to determine BBB permeability during the initial stages of CNS-acting drug design and development (Daneman, 2015; van Tellingen et al., 2015; Saxena et al., 2019).

The BBB separates the CNS from the bloodstream, preventing contagions from invading the brain. Brain endothelial cells, astrocytes, neurons, and pericytes are four major components of the BBB. The largest constituent of the BBB is a layer containing brain endothelial cells, which serve as the first line of defense from the CNS surroundings. Endothelial cells are connected with tight junctions and adherence junctions, which create a strong barrier, restricting pinocytosis and decreasing vesicle-facilitated transcellular transport (Reese and Karnovsky, 1967; Tietz and Engelhardt, 2015). The BBB not only acts as a physical barrier but also serves as a metabolic barrier, transport interface, and secretory layer (Abbott et al., 2006; Rhea and Banks, 2019). Neurons reside very close to brain capillaries and play a vital role in maintaining ion balance in the local environment (Schlageter et al., 1999).

Clinical experiments to determine the BBB permeability of compounds are accurate; however, they are time-consuming and labor-intensive (Bickel, 2005; Massey et al., 2020; Schidlowski et al., 2020). Additionally, it is difficult to perform clinical experiments with diverse types of drug candidates (Main et al., 2018; Mi et al., 2020). Therefore, it is crucial to predict and forecast BBB permeability using computational algorithms or *in vitro* BBB mimics to elucidate the permeability of compounds across the BBB (Gupta et al., 2019). There have been numerous attempts to predict the BBB permeability of compounds since the advent of artificial intelligence (AI), primarily using machine learning (ML) algorithms such as support vector machines (SVMs), artificial neural networks (ANNs), k-nearest neighbors (kNNs), naïve Bayes (NB), and random forests (RFs) (Doniger et al., 2002; Zhang et al., 2008, 2015; Suenderhauf et al., 2012; Khan et al., 2018). In addition to above, some future directions of BBB permeability also seem promising such as application of humanized self-organized models, organoids, 3D cultures and human microvessel-on-a-chip platforms especially those which are amenable for advanced imaging such as transmission electron microscope and expansion microscopy since they enable real-time monitoring of BBB permeability (Wevers et al., 2018; Salman et al., 2020). BBB permeability prediction models developed using AI algorithms can further be assisted with the high throughput screening (Aldewachi et al., 2021), computer aided drug designing (Salman et al., 2021), and knowledge based rules, e.g., Lipinski rule of five (hydrogen bond donor ≤ 5, hydrogen bond acceptor ≤ 10, molecular weight ≤ 500, CLogP ≤ 5), Veber rule (rotatable bonds count ≤ 10, polar surface area ≤ 140), BBB rule (hydrogen bond = 8–10, molecular weight = 400–500, no acids), etc., to screen potential drug candidates with desirable end-point for prevention, mitigation and cure of neurological disorders (Veber et al., 2002; Banks, 2009; Benet et al., 2016).

In an attempt to develop the BBB permeability prediction model, Jiang et al. (2016) applied SVM with a radial basis function (RBF) kernel (Jiang et al., 2016). They used a dataset of 1,562 compounds containing 694 BBB permeable (BBB++) and 868 BBB non-permeable (BBB-) compounds. The overall accuracy, sensitivity, and specificity were reported to be more than 85%. The next year, Castillo-Garit et al. (2017) used the decision tree algorithm on 581 compounds and found that the BBB permeability prediction accuracy increased by 2.93% (Castillo-Garit et al., 2017). However, this study was performed on a much smaller dataset than Jiang et al.’s study. In another study, Yuan et al. (2018) developed SVM-based BBB prediction model using a larger dataset of 1,990 compounds with a prediction accuracy of 93.96% (Yuan et al., 2018). The sensitivity and specificity of the model were reported to be 94.3 and 91.0%, respectively. In the same year, Wang et al. (2018) applied SVM and kNN algorithms using 2,358 compounds (Wang et al., 2018). The prediction accuracy of the best-performing model was found to be 2.64% higher than that of the Yuan et al. (2018) prediction model. However, the model lagged in terms of sensitivity (0.925) and specificity (0.899). The next year, Miao et al. (2019) applied a deep learning (DL) algorithm to 462 compounds. The accuracy of the model was reported to be 97%, with decent AUC (0.98) and F1 scores (0.92). However, the dataset used for the DL study was very small compared to the earlier ML-based models for BBB prediction. Recently, Alsenan et al. (2020) proposed a recurrent neural network (RNN) algorithm-based model using 2,342 compounds for the prediction of BBB permeability (Alsenan et al., 2020). The developed model had better performance metrics with an accuracy, sensitivity, and specificity of 96.53, 94.91, and 98.09%, respectively. The Matthews correlation coefficient (MCC) (93.14) and area under the curve (AUC) (98.6) of the prediction were also found to be satisfactory. In another study, Shaker et al. (2020) applied a light gradient boosting machine algorithm to a dataset of 7,162 compounds for the prediction of BBB permeability (Shaker et al., 2020). Although the study involved a very large dataset compared to previously reported studies, the model’s accuracy was reported to be 90%, which was approximately 6.5% less than the BBB permeability prediction model proposed by Alsenan et al. (2020). In the same year, Singh et al. (2020) used random forest, multilayer perceptron, and sequential minimal optimization using 605 compounds to develop the BBB permeability prediction model (Singh et al., 2020). Upon validation of the developed model using 1,566 compounds, the prediction accuracy was found to be 86.5% only. Very recently, Saxena et al. (2021) proposed an ML-based BBB permeability prediction model using 1,978 compounds (Saxena et al., 2021). The study group found that SVM with the RBF kernel yielded an accuracy of 96.77% with AUC and F1 score values of 0.964 and 0.975, respectively, which outperformed the kNN, random forest, and naïve Bayes algorithms in the prediction of BBB permeability on the same dataset.

The major challenge while applying ML algorithms is selecting optimal features to develop predictive models based on labeled BBB permeability datasets (Hu et al., 2019; Salmanpour et al., 2020). To overcome this challenge, we applied DL algorithms and compared their performance with traditional ML algorithms.

## Materials and Methods

### Data Collection

A total of 3,971 compounds with BBB permeability classes were collected from Zhao et al. (2007); Shen et al. (2010), and Roy et al. (2019). The PubChem database^1^ was used to retrieve available PubChem IDs of the collected compounds. The collected datasets were checked to remove redundant compounds. After careful curation, we obtained a dataset of 3,605 non-redundant clean compounds containing 2,607 BBB permeable and 998 BBB non-permeable compounds (Table 1 and Supplementary File). The class labels for BBB non-permeable and permeable compounds were kept as “0” and “1,” respectively.

**TABLE 1:** The final dataset and its distribution.

Dataset	BBB permeable compounds	BBB non-permeable compounds	Total
Roy et al. (2019)	819	366	1,185
Zhao et al. (2007)	1,398	393	1,791
Shen et al. (2010)	390	239	629
Total	2,607	998	3,605

### Feature Calculation

Three types of feature sets viz. physicochemical properties, molecular access system (MACCS) fingerprints and substructure fingerprints were used in this study. Physicochemical properties contain different types of physical and chemical information encoded in a compound, e.g., molecular weight, molecular volume, solubility, partition coefficient, etc. The molecular fingerprints are fixed-length vectors that indicate the presence/absence of an atom type or functional group in a compound. All features were calculated by open-source PaDel (Yap, 2011). Each compound was encoded with 1,917 features containing 1,444 physicochemical (1D and 2D) properties, 166 MACCS, and 307 substructure fingerprints. This feature set was used for the ML, DNN, and CNN-1D algorithms using Keras framework. For CNN-VGG16, the Python package RDKit was used to generate the structure images of the compounds using their Simplified molecular input line entry system (SMILES) notations (Lovrić et al., 2019; Bento et al., 2020). The Python package RDKit is a collection of ML and cheminformatic software and contains functions to modify chemical compounds. The RDKit package was used to generate 2D images of size 300 * 300 pixels (RGB) from SMILES notations of compounds. RDKit-generated images contain different colors to express the chemical information viz. carbon = black, oxygen = red, nitrogen = blue, sulfur = yellow, chlorine = green, and phosphorous = orange. Images generated by RDKit always fit the entire molecule, so there was no issue with different molecular sizes. The dataset was split into training and test sets at a ratio of 3:1. The test set was separated from the training set to avoid any bias (Table 2). To handle the data imbalance, we have already applied cost-sensitive augmentation via the class_weight argument on the fit() function when training models.

**TABLE 2:** Distribution of the dataset in the training and test sets.

Dataset	BBB permeable compounds	BBB non-permeable compounds	Total
Training set	1,955	749	2,704
Test set	652	249	901
Total	2,607	998	3,605

### Development of Prediction Models

In this study, ML-based algorithms (SVM, kNN, RF, and NB) and DL-based algorithms DNN, CNN-1D were developed using keras framework with libraries; python, numpy, pandas, keras, and tensorflow on Anaconda 3–5.2. CNN (VGG16) was implemented using transfer learning through cloud-based computational resource of Google Colaboratory to develop prediction models for the BBB permeability of the compounds. Based on the performance of the generated prediction models, the DNN-based “DeePred-BBB” is proposed for BBB permeability prediction. DeePred-BBB performance was compared with ML algorithms viz. SVM, NB, kNN, RF, and DL algorithms CNN-1D and CNN (VGG16).

#### Machine Learning-Based Models

Support vector machine with four different kernels (RBF, polynomial, sigmoid, and linear), NB, kNN, and RF were applied to the training set of 2,704 compounds and tested with an independent set of 901 compounds. Principal component analysis (PCA) (Giuliani, 2017) was used for feature reduction. The component range (10, 20, 30, 40, 50, and 100) was used to find the best prediction accuracy for each applied ML algorithm. Tenfold cross-validation was applied to evaluate the efficacy of the model during training.

##### Support Vector Machine

Support vector machine is among the robust ML algorithms used for classification and regression (Ghandi et al., 2014; Gaudillo et al., 2019). It searches for the optimal hyperplane with maximized margins using support vectors for classification (Ben-Hur et al., 2008). This algorithm plots the data to the N-dimensional feature space and finds a hyperplane (Θ.x + b = 0) to classify the data sets with minimized loss using the hinge loss function. The loss function is given in Eq. 1.


(1) $$\left(\theta ,b\right)=\arg\min_{\Theta ,b}\sum_{x\in X}\left[1-y\left(\Theta .x+b\right)\right]+\lambda ||\Theta |{|}_{2}$$

Support vector machine was applied using kernels to map the data to higher dimensions to linearly classify the data (Kumar et al., 2011). A penalty parameter “C” (Cost NAÏVE) adjusts the balance between training errors and forcing rigid margins. Another parameter, “γ,” regulates the kernel function amplitude (Kumar et al., 2018). Various values of C (1, 5, 10, 50, 90) and γ (0.0001, 0.0005, 0.001, 0.005, 0.01, 0.05) were tested to find the best combination. An optimized combination of C and γ was used for each SVM kernel (RBF, *C* = 10, γ = 0.005; polynomial, *C* = 1, γ = 0.005; sigmoid *C* = 90, γ = 0.0005; linear, *C* = 1, γ = 0.05). For the polynomial kernel, 2–6 values of degree (d) were applied and evaluated. The best performance of the polynomial kernel was found at *d* = 3.

##### Naïve Bayes

The naïve Bayes algorithm is based on the Bayes theorem. It is a probabilistic method that works on the assumption of class conditional independence (Eq. 2) (Shen et al., 2019). Each feature present in a class is independent and individually contributes to the probability with nil dependency on other features (Wang et al., 2021a). It is fast, readily manages a large dataset, and generally produces better results than other classification techniques when features existing in a class are independent.


(2) $$P\left(X|Y\right)=\frac{P\left(Y|X\right)P\left(X\right)}{P\left(Y\right)}$$

where P(X|Y) is the posterior probability of X (class) for a given Y (feature), P(Y|X) is the likelihood, P(X) is the prior probability of class X, and P(Y) is the marginal probability of feature Y.

##### k-Nearest Neighbor

k-nearest neighbor is a simple and non-parametric classifier that assumes that nearby data points are similar and tend to have similar classes. Feature similarity is used to find the class label of a new data instance. It commonly uses Euclidean distance to find the closeness of the data points, and depending upon the class matching with considered k-points, the class labels are decided (Sharma et al., 2021). Here, k is the number of neighbors. The Euclidean distance between data points x (x1, x2, x3) and y (y1, y2, y3) is calculated using Eq. 3.


(3) $$d\left(x,y\right)=\sqrt{\left({\left(x_{1}-y_{1}\right)}^{2}+{\left(x_{2}-y_{2}\right)}^{2}+{\left(x_{3}-y_{3}\right)}^{2}\right)}$$

To determine the optimal k, a range of *k*-values (1–10) was evaluated. The best-performing prediction model at *k* = 3 was selected for further analysis.

##### Random Forest

Random forest uses ensemble learning to create a collection of decision trees (forest) that run concurrently and classify data instances (Yao et al., 2020). Tree construction is performed using arbitrary input vectors and node division on arbitrary feature subsets. Each tree of the RF predicts a certain class, and depending upon the highest votes, the final class label is predicted (Yang et al., 2020). In the current study, the developed prediction models were tested with variable trees in a forest (4, 8, 12, 32, 64). Each decision tree’s various depths (2–5) and estimators (5, 10, 20, 30, 40) were tested to find the best performing prediction model.

#### Deep Learning-Based Models

Deep learning algorithms use multiple neurons and hidden layers to extract high-level functions from input data. The major advantage of DL algorithms is their inherent property of selecting the most relevant features from the training dataset. Therefore, unlike ML algorithms, separate feature selection algorithms are not required (Isensee et al., 2021). In this study, three DL algorithms, DNN, CNN-1D, and CNN-VGG16, were applied. The tenfold cross-validation method was used to assess the model’s efficiency while training. The training dataset was further divided into ten subsets, iteratively training models using all subsets except one held out to test the performance.

##### Deep Neural Network

For DNN, 2,704 compounds, each encoded with 1,917 features (1,444 physicochemical properties, 166 MACCS, and 307 substructure fingerprints), were used to develop BBB permeability prediction models. Initial layers receive compounds encoded with feature vectors and subject them to the hidden layers. These hidden layers obtain the relevant information from the input vectors and project the freshly extracted features to the batch normalization layer. This layer increases the training process by reducing the intradata covariance. Dropout layers were applied to reduce the problem of coadaptation of neurons and overfitting (Baldi and Sadowski, 2014). These layers randomly drop the nodes as per the dropout rate. Rectified linear unit (ReLU) activation function was used, which adaptively transforms rectifier parameters. Furthermore, ReLU transforms the neuronal output by mapping it to the highest possible value or zero (if the value is negative) (Wang et al., 2021b). ReLU function is given in Eq. 4.


(4) F(xi)=max(0,xi)

where xi is input for activation function f on channel “i.”

The “softmax” activation function was applied on the output layer to map the hidden layer output between 0 to 1 intervals. The Adam optimizer was used to minimize the loss value from the cross-entropy cost function.

The network performance of a DNN depends upon its depth and breadth. Therefore, it is vital to determine the optimal depth and breadth and optimize other parameters, e.g., the learning rate and dropout ratio. To achieve this, we kept other parameters fixed and evaluated the prediction accuracy by varying the hidden layers (*K* = 1–5) and neurons (100, 200, 300, 500, 800 neurons per layer). The DNNs were also simultaneously evaluated for five dropout ratios (0.1, 0.2, 0.3, 0.4, 0.5), and prediction accuracy was evaluated. Furthermore, various network configurations were evaluated for epochs (100, 200, 400, 500, 800) and learning rates (0.0001, 0.0002, 0.0003, 0.001, 0.002, 0.003) optimization. Table 3 summarizes the explored values of hyperparameters for the development of the DNN-based BBB permeability prediction model.

**TABLE 3:** Hyperparameter values explored for the DNN model.

Parameter	Values
Number of hidden layers	1–5
Number of neurons	100, 200, 300, 500, 800
Dropout ratio	0.1, 0.2, 0.3, 0.4, 0.5
Learning rate	0.0001, 0.0002, 0.0003, 0.001, 0.002, 0.003
Epochs	100, 200, 400, 500, 800

##### Convolutional Neural Network-1 Dimension (CNN-1D)

CNN is a particular type of DL that is widely used for image data classification (LeCun et al., 2015). There are three major layers in the CNN: convolutional, pooling, and fully connected layers. Cube-shaped weights and multiple filters (kernels) are applied in the convolutional layers to extract features and develop feature maps from the images (Esteva et al., 2017; Malik et al., 2021; Shan et al., 2021). The filter size may downsample the outputs; therefore, the size and number of kernels are vital (D’souza et al., 2020). To overcome the issue of downsampling, an optimized padding value is applied, which allows the filter kernels to create feature maps of the input image size.

Furthermore, other parameters of the convolutional layer also needed to be optimized, e.g., regularization type and value, activation function, and stride. Pooling layers specifically perform average or max-pooling in the filter region to lower the number of parameters and calculations by downsampling the representations. The fully connected layers flatten the output prior to the classification and are usually kept at the end. CNNs are created to process and learn from images. However, CNN-1D can be applied similarly to one-dimensional data containing physicochemical properties and fingerprints. We used three filters (15, 32, 64) to determine the local pattern in the 1,917 features, which were calculated from PaDel. After the CNN layers, dense layers (1 and 2) were tested for three dropout ratios (0.2, 0.3, 0.5). Table 4 summarizes the explored hyperparameter values for the development of the CNN-1D model.

**TABLE 4:** Explored hyperparameter values for the CNN-1D model.

Parameter	Values
Number of filters	15, 32, 64
Number of dense layers	1, 2
Dropout ratio	0.2, 0.3, 0.4
Learning rate	0.0001, 0.0002, 0.0003, 0.001, 0.002, 0.003
Epochs	100, 200, 400, 500, 600

##### Convolutional Neural Network by VGG16 Transfer Learning (CNN-VGG16)

The CNN processes the input 2D images to distinguish the image objects by allocating weights and biases. CNN captures temporal and spatial relationships using the tiny squares of input images by processing them through a series of convolution layers. Filters in each convolutional layer skid on the image to find relevant and specific features, e.g., edge detection, sharpen or blur the image and produce the feature map. The feature map’s size depends on filter numbers, filter slide-over pixels, and zero-padding (image borders are padded with zero). The 2D-array values of the feature map were subjected to the individual layer activation function (ReLU). Dimensionality reduction of each feature map is processed using pooling without any loss of information. The pooling layer’s output is sent into fully connected layers, which classify the images. The CNN with transfer learning (VGG16) was used in this study using RDKit-generated images. The images were scaled to a pixel size of 128 * 128 to develop and validate the BBB permeability prediction model.

**FIGURE 2:**
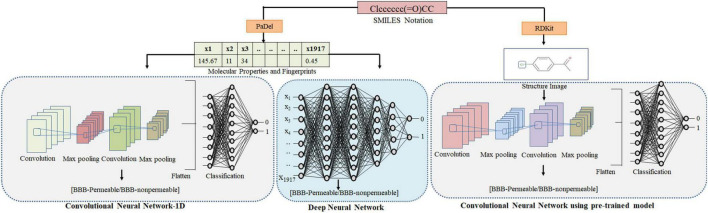
Methodology adopted for predicting the BBB permeability of compounds using SMILES notation. The SMILES notations were used to calculate molecular properties and fingerprints using PaDel. These features were used as input to DNN and CNN-1D to generate the BBB permeability prediction models. The 2D images of compounds were generated using RDkit and fed to the CNN (VGG16) to generate the BBB permeability prediction model.

Furthermore, image data argumentation was performed by randomly zooming (up to 10%) and flipping the images. The CNN (VGG16) hyperparameters are given in Table 5. The developed model was tested with an independent test set consisting of 901 images. Figure 2 depicts the adopted methodology to develop the DL-based prediction models.

**TABLE 5:** The hyperparameters for CNN (VGG16).

Parameters	VGG16
Convolutional blocks	Convolutional layers, Kernel size, Filters, Max-Pooling, Zero Padding: Predefined
Dense layers	02
Dense layers neurons	150, 104
Dropout ratio	0.5
Learning rate	0.02
Batch size	132
Epochs	800

## Results and Discussion

The performance metrics of the ten developed models (ML = 7, DL = 3) for BBB permeability prediction were compared to determine the best-performing model. The performance indicators used in this study were area under the curve (AUC), area under the precision-recall curve (AUPRC), average precision (AP), F1 score and accuracy, and Hamming distance (HD) of the prediction models. Among the developed ML prediction models, the SVM (RBF kernel)-based prediction model outperformed the NB, kNN, and RF algorithms for BBB permeability prediction with test set data. The accuracy of SVM (RBF) was found to be approximately 6% higher than that of NB and RF and approximately 1% higher than that of kNN. Moreover, SVM (RBF) yielded better prediction values of other performance indicators in BBB permeability prediction on the given dataset. However, the performance metrics of SVM (polynomial) at degree 3 were found to be very comparable to the SVM (RBF).

**FIGURE 3:**
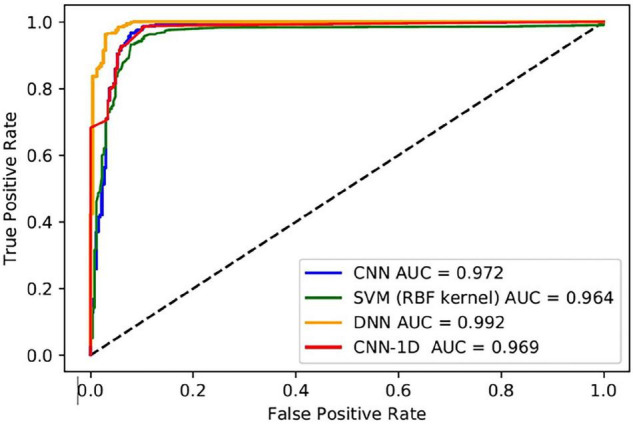
ROCs of the best performing models (CNN, SVM, DNN, and CNN-1D) and their respective AUCs.

**FIGURE 4:**
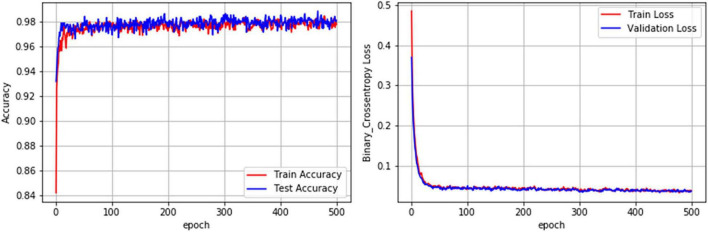
Accuracy and loss plot of the DNN model for the prediction of BBB permeability.

The performance metrics of the DL algorithms were found to be very close to each other. The prediction accuracies of DNN, CNN-1D, and CNN (VGG16) were 98.07, 97.44, and 97.66, respectively. However, the DNN model was superior in AUC, AUPRC, AP, F1, and HD when compared to that of CNN-1D and CNN (VGG16) (Table 6). The comparison of receiver operating characteristic (ROC) curves between SVM (RBF), DNN, CNN-1D, and CNN (VGG16) also indicates the superiority of DNN in BBB permeability prediction with the given dataset (Figure 3). Furthermore, the accuracy and loss plots of the DNN model are given in Figure 4. The accuracy plot shows good coherence between the training (red) and test (blue) accuracy, suggesting that the model is not overfitted. Additionally, coherence in the training (red) and validation/test (blue) loss in the loss plot (binary cross-entropy loss) is indicative of an unbiased model (Figure 4).

**TABLE 6:** Performance metrics of ML and DL algorithms.

Algorithm	AUC	AUPRC	AP	F1	A (%)	HD	FPR (%)	FNR (%)
SVM (RBF)	0.964	0.988	0.975	0.985	96.29	0.022	6.451	0.724
SVM (Polynomial *d* = 3)	0.948	0.965	0.965	0.98	96.01	0.029	9.756	0.579
SVM (Sigmoid)	0.921	0.971	0.944	0.962	94.45	0.055	13.359	2.519
SVM (Linear)	0.916	0.969	0.938	0.963	94.56	0.054	15.242	1.497
NB	0.844	0.948	0.899	0.935	90.18	0.098	14.543	3.202
kNN (3)	0.927	0.974	0.949	0.968	95.3	0.047	12.891	1.615
RF (3, 20)	0.815	0.943	0.887	0.938	90.29	0.0971	26.666	1.471
**DNN**	**0.992**	**0.997**	**0.996**	**0.987**	**98.07**	**0.019**	**4.048**	**1.159**
CNN-1D	0.969	0.956	0.975	0.983	97.44	0.026	4.118	2.017
CNN (VGG16)	0.972	0.983	0.983	0.946	97.61	0.0804	4.581	2.326

*AUC, area under curve; AUPRC, area under precision-recall curve; AP, average precision; F1, F1 score; A, accuracy; HD, hamming distance; FPR, false positive rate; FNR, false negative rate; SVM, support vector machine; RBF, radial basis function; d, degree; NB, naïve Bayes; kNN, k-nearest neighbor; RF, random forest; DNN, deep neural network; CNN-1D, convolution neural network-one dimension; CNN (VGG16), convolution neural network- visual geometry group16. Best performing model (highlighted in bold).*

The better performance of DL algorithms compared to ML could be due to their ability to handle the large dataset and extract the most relevant features of their own. The performance metrics of DNN, CNN-1D, and CNN (VGG16) were very comparable. To our surprise, DNN was found to be slightly better in overall performance based on accuracy and other performance indicators compared to the CNN models. DNN appears to be a better option to handle the compounds encoded with physicochemical and fingerprint features for classifications and predictions. Based on the overall performance, we selected the DNN model for the development of “DeePred-BBB.” DeePred-BBB can predict BBB permeability based on chemical SMILES notation. It uses PaDel to calculate the features from the SMILES notation and sends them as input to the DNN model. The output is either permeable or non-permeable.

Compounds can penetrate the BBB using various different mechanisms, such as transmembrane diffusion, adsorptive endocytosis, saturable transporters, and extracellular pathways. Most drugs in clinical use till date are small, lipid soluble molecules that cross the BBB by transmembrane diffusion. The prediction models to determine the mechanism of BBB permeability require a mechanism-based set of compounds with their permeability class labels for each mechanism. However, the current study deals with the prediction of the BBB permeability of compounds (irrespective of how they penetrate the BBB) using their SMILES notations. The study holds limitations in identifying the mechanism by which compounds are BBB permeable. DeePred-BBB does not take multiple SMILES notations for prediction. The user needs to input the SMILES notations of compounds one at a time for accurate prediction of BBB permeability. A comparison between DeePred-BBB and previously reported BBB permeability prediction models is given in Table 7.

**TABLE 7:** Comparative analysis of DeePred-BBB with recently published BBB permeability prediction models.

Algorithm	Data set	Prediction performance	Study group
SVM (RBF)	1,562 compounds (BBB+ = 694, BBB- = 868)	>85% accuracy, sensitivity, and specificity	Jiang et al., 2016
Decision trees	581 compounds	Accuracy = 87.93%, Sensitivity = 86.67% Specificity = 89.29%	Castillo-Garit et al., 2017
SVM (RBF)	1,990 compounds (BBB permeable = 1,550, BBB non-permeable = 440)	Accuracy = 93.96%, Sensitivity = 94.3%, Specificity = 91.0%, MCC = 0.84	Yuan et al., 2018
SVM, kNN	2,358 compounds	Accuracy = 96.6%, Sensitivity = 92.5%, Specificity = 89.9%	Wang et al., 2018; Mi et al., 2020
DL	462 compounds (BBB permeable = 250, BBB non-permeable = 212)	Accuracy = 97%, AUC = 0.98, F1 = 0.92	Miao et al., 2019
RNN	2,342 compounds	Accuracy = 96.53%, Sensitivity = 94.91%, Specificity = 98.09%, MCC = 0.931, AUC = 0.986	Alsenan et al., 2020
Light Gradient Boosting Machine Algorithm	7,162 compounds (BBB permeable = 5,453 BBB non-permeable = 1,709)	Accuracy = 90%, sensitivity = 85%, specificity = 94%	Shaker et al., 2020
RF, Multilayer perceptron, Sequential minimal optimization	605 compounds (training) +1,566 compounds (validation)	Accuracy = 86.5%	Singh et al., 2020
SVM (RBF)	1,978 compounds (BBB permeable = 1,550, BBB non-permeable = 440)	Accuracy = 96.77%, AUC = 0.964, F1 = 0.975	Saxena et al., 2021
DNN	3,605 compounds (BBB permeable = 2,704 BBB non-permeable = 901)	Accuracy = 98.07%, AUC = 0.992, AP = 0.997, F1 = 0.987	Current study

*AUC, area under curve; AUPRC, area under precision-recall curve; AP, average precision; BBB, blood brain barrier; DL, deep learning; F1, F1 score; kNN, k-nearest neighbor; MCC, Matthews correlation coefficient; RBF, radial basis function; RF, random forest; RNN, recurrent neural network; SVM, support vector machine.*

## Conclusion

Deep learning and machine learning algorithms were applied to a dataset of 3,605 compounds to develop a prediction model that could accurately predict the BBB permeability of compounds using their SMILES notations as input. The comparative analysis of the performance metrics of the developed models suggested that the overall performance of DNN-based BBB permeability prediction is better than that of the ML and CNN models. It was discovered that the notion of “deeper the network, better the accuracy” does not often hold true. An optimal depth of the network is required beyond which the performance of the network does not improve. A DNN model with three layers (depth) having 200, 100, and 2 nodes each was the most accurate. It was also observed that in the case of compounds, the physicochemical properties and fingerprint-based DL models yield slightly better performance than 2D-structure image-based models in BBB permeability prediction.

Based on this study, we propose the DeePred-BBB model for BBB permeability prediction of compounds using their SMILES notations as input. In DeePred-BBB, the best performing DNN model is integrated with the open-source PaDel tool to calculate features. The calculated features are automatically fed to the DNN model as input, which predicts whether the compound will be BBB permeable or non-permeable. DeePred-BBB could assist in making quality decisions regarding which compound to carry forward in subsequent drug development stages and could potentially help in reducing the attrition rate of CNS-acting drug candidates failing due to BBB non-permeability. Inevitably, such drug candidates need further *in vivo* validation to arrive at efficacious and safe drugs at a faster rate and lower cost. DeePred-BBB could be accessed at https://github.com/12rajnish/DeePred-BBB.

## Data Availability Statement

The original contributions presented in the study are included in the article/Supplementary Material, further inquiries can be directed to the corresponding author/s.

## Author Contributions

RK and AS access to all of the data analyzed in this study, drafted the manuscript, and performed the statistical analysis. RK takes responsibility for the integrity and accuracy of the study data analysis and results. RK, AS, AA and GA involved in the study design, concept, analysis, and interpretation of data. AB, MK, AA, and GA involved in critical revision of the manuscript. All authors contributed to the article and approved the submitted version.

## Conflict of Interest

The authors declare that the research was conducted in the absence of any commercial or financial relationships that could be construed as a potential conflict of interest.

## Publisher’s Note

All claims expressed in this article are solely those of the authors and do not necessarily represent those of their affiliated organizations, or those of the publisher, the editors and the reviewers. Any product that may be evaluated in this article, or claim that may be made by its manufacturer, is not guaranteed or endorsed by the publisher.

## Footnotes

1
https://pubchem.ncbi.nlm.nih.gov/
